# Genome-Wide Association Study in Bipolar Patients Stratified by Co-Morbidity

**DOI:** 10.1371/journal.pone.0028477

**Published:** 2011-12-21

**Authors:** Berit Kerner, Christophe G. Lambert, Bengt O. Muthén

**Affiliations:** 1 Department of Psychiatry, David Geffen School of Medicine, University of California Los Angeles, Los Angeles, California, United States of America; 2 Golden Helix Incorporated, Bozeman, Montana, United States of America; 3 Graduate School of Education and Information Studies, Social Research Methodology Division, University of California Los Angeles, Los Angeles, California, United States of America; University of Wuerzburg, Germany

## Abstract

**Background:**

Bipolar disorder is a severe psychiatric disorder with high heritability. Co-morbid conditions are common and might define latent subgroups of patients that are more homogeneous with respect to genetic risk factors.

**Methodology:**

In the Caucasian GAIN bipolar disorder sample of 1000 cases and 1034 controls, we tested the association of single nucleotide polymorphisms with patient subgroups defined by co-morbidity.

**Results:**

Bipolar disorder with psychosis and/or substance abuse in the absence of alcohol dependence was associated with the rare variant rs1039002 in the vicinity of the gene phosphodiesterase 10A (*PDE10A*) on chromosome 6q27 (p = 1.7×10^−8^). *PDE10A* has been implicated in the pathophysiology of psychosis. Antagonists to the encoded protein are currently in clinical testing. Another rare variant, rs12563333 (p = 5.9×10^−8^) on chromosome 1q41 close to the MAP/microtubule affinity-regulating kinase 1 (*MARK1*) gene, approached the genome-wide level of significance in this subgroup. Homozygotes for the minor allele were present in cases and absent in controls. Bipolar disorder with alcohol dependence and other co-morbidities was associated with SNP rs2727943 (p = 3.3×10^−8^) on chromosome 3p26.3 located between the genes contactin-4 precursor (*BIG-2*) and contactin 6 (*CNTN6*). All three associations were found under the recessive genetic model. Bipolar disorder with low probability of co-morbid conditions did not show significant associations.

**Conclusion:**

Conceptualizing bipolar disorder as a heterogeneous disorder with regard to co-morbid conditions might facilitate the identification of genetic risk alleles. Rare variants might contribute to the susceptibility to bipolar disorder.

## Introduction

Bipolar disorder (BPD) is a mental disorder with dramatic and unpredictable mood swings between mania and depression. It affects approximately 5.7 million American adults or 2.6 percent of the U.S. population aged 18 and older in a given year (http://www.nimh.nih.gov/health/publications/the-numbers-count-mental-disorders-in-america/index.shtml). The symptoms of BPD vary considerably. Therefore, this disorder has also been conceptualized as a group of related mood disorders referred to as bipolar spectrum disorders. It remains unclear if these conditions share a common pathophysiology or common risk factors. Recently, co-morbid conditions in BPD have drawn attention as potential indicators of pathologically distinct subtypes [1].

Co-morbid conditions are common in BPD. They are often related to the severity of the disorder and may aggregate in families. Familial aggregation has been shown for co-morbid attention deficit hyperactivity disorder (ADHD) [2], alcohol use disorders, and panic disorder [3], as well as psychosis [4]. Familial aggregation of co-morbid conditions can be caused by shared environment, shared genetic risk factors or a combination of both. Therefore, co-occurrence in families alone cannot clearly differentiate between these possible causes. Twin studies comparing the occurrence rate in monozygotic twins versus dizygotic twins revealed that the susceptibility to BPD, substance abuse, as well as alcohol dependence could be influenced by genetic factors [5]–[9]. Shared pathophysiology might explain the common and characteristic co-occurrence of these disorders [10], [11]. Exposure to substances of abuse at certain vulnerable times during development might in itself increase risk for psychotic disorders in individuals with certain genetic susceptibilities [12], [13]. The identification of genetic risk alleles associated with BPD, substance abuse, or a combination of both might assist in disentangling these complex and interrelated factors.

The search for genetic risk factors in BPD has recently drawn attention to some genomic variants that might play a role in BPD. In genome-wide association studies, several common variants have been found to be significantly associated with BPD. Among those are the single nucleotide polymorphism (SNP) rs1012053 in the first intron of the gene diacylglycerol kinase eta (*DGKH*) (HGNC:2854) on chromosome 13q14.1 [14], variants in the gene voltage-dependent L-type calcium channel subunit alpha-1C (*CACNA1C*) (HGNC:1390) on chromosome 12p13 [15] and the intronic SNP rs10994336 in the Ankyrin-3 (*ANK3*) (HGNC:494) gene on chromosome 10q21 [16], [17]. In addition, rare structural variants as well as common variants in gene deserts have been suggested. However, replication of these findings has been challenging. A previous analysis of the data set used in our analysis failed to report genome-wide significant associations [18].

BPD is likely to be a heterogeneous disorder. Samples consisting of unidentified subgroups that behave differently with regard to the problem at hand introduce noise to the data and might lead to increased type 1 and type 2 errors [19]. In order to explore phenotype heterogeneity in the Genetic Association Information Network (GAIN) Bipolar Disorder sample, we used a multivariate latent class analysis (LCA) to define subgroups of BPD patients based on profiles of psychiatric co-morbid conditions. Using this approach, BPD patients could be assigned to their most likely latent class. We then tested the relationship of SNPs from a genome-wide scan genotyped on the Affymetrix 6.0 array for association with the latent classes. Our approach identified several highly significant associations with rare, as well as common, genomic variants.

Subgroups of patients defined by co-morbid conditions might be more homogeneous with respect to underlying genetic risk factors. Therefore, sub-grouping of BPD patients according to co-morbid conditions might identify additional genomic variants that are associated with a particular sub-phenotype.

## Materials and Methods

### Ethics statement

All participants gave informed consent for the inclusion in genetic studies on BPD. Written consent was given by the patients for their information to be stored in the National Institute of Heath database and used for research. No identifiable data were used in this study. Only completely de-identified data had been made available to the researchers involved in this study. The study had been exempt from institutional review by the Institutional Review Board at the University of California, Los Angeles, based on the fact that only preexisting and completely de-identified data were analyzed.

### Sample

The BPD sample consisted of 1041 unrelated individuals from the Foundation for the National Institutes of Health Genetic Association Information Network (GAIN) Initiative (http://www.genome.gov/19518664) [20]. Individuals were of European descent according to self-reported heritage. The sample has been used in several previously published studies [15], [18], [21]. Best-estimate diagnosis procedures had been used to diagnose mood disorder, as well as co-morbid conditions according to the Diagnostic and Statistical Manual of Mental Disorder Version III Revised (DSM-III-R) and DSM-IV diagnostic criteria [22]. Information on mood symptoms, psychotic symptoms and co-morbid conditions was obtained based on the Diagnostic Interview for Genetic Studies (DIGS) Version 3 and 4 (http://www.nimhgenetics.org) [23], family information and medical records. Multiple diagnoses per individual were allowed if diagnostic criteria were fulfilled for each disorder independently. Information about lifetime symptoms of hallucinations and/or delusions was obtained through the K section of the DIGS. Based on these information sources forty-one subjects did not meet full DSM-IV criteria for BPD or schizoaffective disorder and therefore, they were subsequently excluded from the analysis. The remaining 1000 individuals, all of whom were diagnosed with BPD type I, were included in the latent class analysis and the genetic association study. The cases consisted of 499 males and 501 females. The age at interview ranged from 17 to 88 years. The mean age was 40.1 years, with a standard deviation (SD) of 12.6 years (Table 1). Age was missing in 8 individuals, 5 males and 3 females.

**Table 1:** Demographic description of the bipolar disorder sample and controls.

	Controls	BPD	Latent Class 1	Latent Class 2	Latent Class 3
	N = 1034	N = 1000	N = 258	N = 249	N = 493
	100%	100%	26%	25%	49%
**AGE (mean)**	52 (SD = 17.6)	42# (SD = 12.6)	41 (SD = 12.6)	41 (SD = 10.8)	44* (SD = 13.8)
**MAX**	90	88	64	80	88
**MIN**	18	17	18	17	18
**Female**	502 (49%)	501 (50%)	160 (62%)*	96 (39%)*	240 (49%)*
**Male**	532 (51%)	499 (50%)	98 (38%)	153 (61%)	248 (51%)
**BPD**	0 (0%)	1000 (100%)	258 (100%)	249 (100%)	493 (100%)
**BPD+SUB**	0	336 (34%)	111 (43%)	200 (80%)	25 (5%)
**BPD+OCD**	0	82 (8%)	50 (19%)	24 (10%)	8 (2%)
**BPD+PD**	0	236 (24%)	97 (38%)	77 (31%)	62 (13%)
**BPD+SP**	0	161 (16%)	64 (25%)	57 (23%)	40 (8%)
**BPD+ED**	0	70 (7%)	37 (14%)	25 (10%)	8 (2%)
**BPD+ADHD**	0	97 (10%)	43 (17%)	50 (20%)	4 (1%)
**BPD+ALCAB**	0	134 (13%)	133 (52%)	1 (0.4%)	0 (0%)
**BPD+ALCDEP**	0	332 (33%)	0 (0%)	249 (100%)	83 (17%)
**BPD+NIC**	0	241 (24%)	90 (35%)	125 (50%)	26 (5%)
**BPD+PSYCH**	0	252 (25%)	105 (41%)	68 (27%)	79 (16%)

# Age was missing in 8 individuals, 5 males and 3 females, all of whom clustered into Latent Class 3.

*Significant at the 0.05 level in the Wald Chi-Square test when testing the equality of means across the latent classes.

SUBA, substance abuse; OCD, obsessive compulsive disorder; PD, panic disorder; SP, social and specific phobia; ED, eating disorder; ADHD, attention-deficit hyperactivity disorder; ALCAB, alcohol abuse; ALCDEP, alcohol dependence; NIC, nicotine dependence; PSYCH, psychotic symptoms (presence of hallucinations and/or delusions).

**Figure 1:**
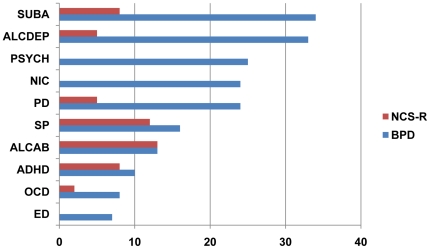
Life-time prevalence of co-morbid conditions and psychotic symptoms in bipolar patients of the GAIN study compared to lifetime prevalence in the National Comorbidity Survey Replication Study [**51**]. Co-morbid conditions and psychotic symptoms are common in bipolar disorder. In the GAIN sample of 1000 bipolar patients, substance abuse/dependence was the most prevalent co-morbid condition, followed by alcohol dependence. Nicotine dependence was present in about 25% of individuals. The prevalence of panic disorder was consistent with other reports in the literature [23]. Attention deficit hyperactivity disorder, obsessive-compulsive disorder and eating disorder were generally rare, present in less than 7% of individuals. As a comparison we used lifetime prevalence rates of psychiatric disorders from the National Comorbidity Survey Replication Study (NCS-R). In this study the evaluation of ADHD was limited to a subsample of individuals age 44 and younger, which might explain the high prevalence rate. Psychotic symptoms, nicotine dependence and eating disorders had not been evaluated in this study. SUBA, substance abuse; ALCDEP, alcohol dependence; PSYCH, psychotic symptoms (presence of hallucinations and/or delusions); NIC, nicotine dependence; PD, panic disorder; SP, social and specific phobia; ALCAB, alcohol abuse; ADHD, attention-deficit hyperactivity disorder; OCD, obsessive compulsive disorder; ED, eating disorder.

Co-morbid conditions were prevalent in the BPD patients (Table 1, Figure 1). Conditions present in at least 5% of individuals with BPD were used as indicators of the latent subclasses. Ten variables fulfilled this criterion: substance abuse or dependence (SUBA) (336 individuals), alcohol dependence (ALCDEP) (332 individuals), nicotine dependence (NIC) (241 individuals), panic disorder (PD) (236 individuals), social phobia and specific phobia (SP) (161 individuals). Alcohol abuse (ALCAB) (134 individuals), attention deficit hyperactivity disorder (ADHD) (97 individuals), obsessive compulsive disorder (OCD) (82 individuals), and eating disorder (ED) (70 individuals) were only slightly more frequent than in the general population [24]. Presence of psychotic symptoms, such as hallucination and/or delusions (PSYCH) (252 individuals) were also evaluated and included in the analysis, even though we did not consider them as a co-morbid disorder, but rather a part of the bipolar phenotype. Prior evidence suggested that these symptoms might indicate a particular subtype of BPD [25].

The controls in the GAIN study had been ascertained independently through an NIMH funded initiative. Originally, a total of 4,586 subjects across the U.S. had been asked to complete a psychiatric and medical questionnaire. Based on the response, only individuals who did not fulfill diagnostic criteria for major depression, psychosis or BPD were included as controls. Out of this pool, one thousand and thirty-four control samples matched for gender and ethnicity (532 males and 502 females) were selected as controls in the GAIN sample. The age at interview ranged from 18 years to 90 years with a mean age of 52.5 years and a SD of 17.6 years. Since individual-level information on psychiatric conditions and symptoms was not available for the controls, this information could not be included in the latent class model. Our analysis is therefore limited to the evaluation of co-morbid conditions in the context of BPD only. The majority of cases and controls were older than the average age of onset for BPD or the co-morbid conditions, but since information on age was not available on the individual level, uncertainties in some controls could not be completely excluded. Since this study is a reanalysis of publicly available data, these design issues were beyond our control.

### Latent class analysis

The LCA was performed in the statistical software program Mplus [26]. The latent class membership was estimated by maximum likelihood using the estimation maximization (EM) algorithm [27]. We used 1000 random sets of starting values to avoid local maxima in the log-likelihood. Missing data was handled by including all available observations in line with the assumption of “missing at random” (MAR) [28]. This assumption allows for missingness to be a function of covariates in contrast to the assumption “missing completely at random”. The only variable with missing data was age at interview. The Bayesian information criterion (BIC) was calculated for the different class solutions, where the model with the smallest BIC was selected as the best [29].

In this latent class framework, 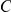 denotes a latent variable, and 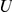 stands for the binary, categorical or count observed indicator variables. Let 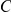 denote a latent categorical variable with 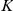 classes, 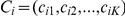, where 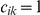 if individual 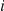 belongs to class 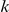 and zero otherwise. For 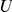, conditional independence is assumed given 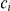,
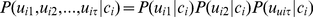
(1)As a first step we conducted the latent class analysis without any covariates in the model in order to understand the substantive interpretation of the latent classes. Then we included sex and age as auxiliary variables in the model. Conditional class specific means were evaluated for each auxiliary variable based on the estimated latent class model (option e in Mplus). We then tested the equality of means across the latent classes using the Wald Chi-Square test based on draws from the posterior probabilities. The magnitude of the mean differences between the classes was interpreted as an indicator for the strength of the prediction that the auxiliary variable influenced the class membership [30]–[32].

### Genotyping and quality control

Genotyping was carried out by the Broad Institute Center for Genotyping and Analysis using the Affymetrix Genome-Wide Human SNP Array 6.0 (Affymetrix, Santa Clara, CA, USA) separately for cases and controls to facilitate sharing of controls with the Genome-Wide Association Study of Schizophrenia (dbGaP; Study Accession: phs000021.v2.p1). The identity of the samples including gender identity had been checked at Rutgers University by genotyping a 24-SNP panel on the Sequenom iPLEX platform. A detailed description of the extensive quality control of the data before public release can be found elsewhere [20]. Even after those quality control measures, significant batch effects due to non-randomization of cases and controls were present in the publicly available data.

Therefore, in this analysis, we recalled the genotypes on the raw signal intensity measurements of the data using the corrected robust linear model with the maximum likelihood distance (CRLMM) algorithm and the BEAGLECALL algorithm [33], [34]. CRLMM used the Oligo 1.6.0 package from Bioconductor 2.3. (http://www.bioconductor.org /download/oldrelease/BioC2.3/). All samples were run together in a single computational batch using default parameters. Recently the BEAGLECALL methodology was introduced to improve the accuracy of genotype calls through the use of haplotype phase information and linkage disequilibrium (LD) structure [35]. We used BEAGLECALL version 0.9.4, which invokes the haplotype phasing methods of BEAGLE version 3.1. [36]. BEAGLECALL requires a matrix of genotype probabilities for the 3 genotypes as well as normalized A and B allele intensities. CRLMM calls were used as a starting point for BEAGLECALL – all calls were made regardless of confidence, and the initial probabilities for each call were set to one, with the other two genotype probabilities set to zero. Autosomal allele intensities were extracted from the raw CEL files and quantile normalized [37] using Golden Helix SNP & Variation Suite (SVS) software (http://www.goldenhelix.com). Three iterations of BEAGLECALL were run with the recommended call thresholds of 0.8 for the first iteration, 0.96 for the second and 0.97 for the third. We used the default BEAGLECALL Hardy-Weinberg quality control threshold of 10^−6^. Genotype calls with confidence less than 0.97 were set as missing. Our analysis was limited to autosomal chromosomes only, since the BEAGLECALL software currently does not support genotype calls for X and Y chromosomal markers in male individuals. Starting with 868,157 autosomal calls from CRLMM, BEAGLECALL provided 796,664 high quality autosomal SNP calls. After eliminating SNPs with minor allele frequency (MAF)<0.001, the final dataset consisted of 728,331 autosomal SNPs. When association studies were performed across all combined samples with the case/control status, the Q-Q plots and Manhattan plots indicated little evidence of spurious associations due to batch effects (File S1). A total of 1,000 cases and 1,034 controls had genotypes and phenotypes that met quality control standards and those were included in the genetic analysis using the latent class membership probability as phenotype. We tested for association under the dominant, additive and recessive model. Association analyses were performed with the SVS software from Golden Helix. Correction for population stratification and outliers in the genotype data was performed with principal component analysis [38]. Q-Q plots were examined for each analysis (File S1) and cluster plots were manually examined for all significant findings. Genome-wide significant results were retested with 1,000 permutations. In addition, we re-genotyped all genome-wide significant SNPs with the TaqMan® SNP Genotyping Assay from Applied Biosystems run on the ABI 7900 Fast Real-Time PCR System (Life Technologies Corporation, Carlsbad, CA, USA) according to the published protocol (http://www3.appliedbiosystems.com/cms/groups/mcb_support/documents/generaldocuments/cms_042998.pdf). The genome browser used for the bioinformatics analysis was the Ensemble database, assembly GRCh37.p2, Feb 2009, Version 60.37e.

## Results

### Prevalence of co-morbid conditions

In the GAIN sample ascertained for genetic studies on bipolar disorder, co-morbid conditions were prevalent. The frequency of alcohol dependence, substance abuse and panic disorder in the GAIN sample reflected the well-known and characteristic co-morbidity in bipolar disorder that has emerged in recent studies in the U.S., as well as worldwide. In this regard, this relatively small sample was very similar to larger population samples of bipolar disorder published in recent years [24], [39]. However, the sample differed from published data in the frequency of specific and social phobias, ADHD and OCD, which were only slightly higher than in the general population. The prevalence of alcohol abuse was comparable to the prevalence in the National Comorbidity Survey Replication Study (NCS-R) (). In order to evaluate possible clustering of co-morbid conditions and unobserved subgroups of individuals characterized by co-morbidity profiles, we performed a latent class cluster analysis.

### Latent class analysis

**Figure 2:**
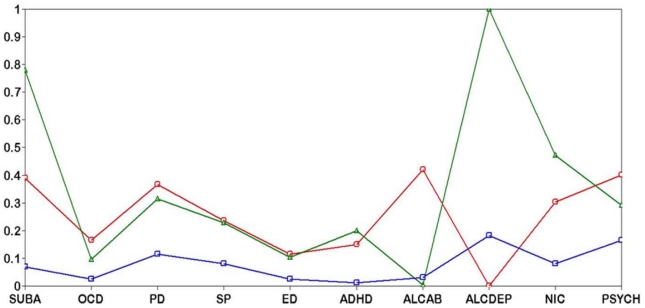
Latent class solution of co-morbid conditions in bipolar disorder (BPD). Co-morbid conditions might indicate heterogeneity in BPD. Latent class mixture modeling is a multivariate statistical approach to heterogeneity in data. The figure shows the co-morbidity profile of the three latent classes of BPD patients. Latent Class 1 (red line and ○ symbol) consisted of BPD patients with substance abuse and/or psychosis and a low probability of endorsing alcohol dependence. Class 2 (green line and Δ symbol) was BPD patients with a high probability of endorsing substance abuse and alcohol dependence. The probability of endorsing nicotine dependence in this class was the highest of all latent classes. Regarding obsessive compulsive disorder, panic disorder, social phobia, eating disorder, and attention-deficit hyperactivity disorder, Class 1 and Class 2 were not clearly differentiated. Individuals in Class 3 (blue line and □ symbol) were diagnosed with BPD, but their probability of endorsing any co-morbid condition was very low. The x-axis indicates the co-morbid conditions included in the LCA. The y-axis represents the probability of endorsing co-morbid conditions scaled from 0% to 100%. SUBA, substance abuse; OCD, obsessive compulsive disorder; PD, panic disorder; SP, social and specific phobia; ED, eating disorder; ADHD, attention-deficit hyperactivity disorder; ALCAB, alcohol abuse; ALCDEP, alcohol dependence; NIC, nicotine dependence; PSYCH, psychotic symptoms (presence of hallucinations and/or delusions).

In the latent class analysis, subgroups of patients with significant differences in co-morbidity profiles became apparent. A three-class solution was selected as indicated by the Bayesian information criterion (BIC) (Figure 2, Table 1). The entropy of the 3-class solution was 0.694, indicating a fair separation of the latent classes. Class 1 (26% of the sample) was BPD patients, who had a high probability of endorsing substance abuse and psychotic symptoms. Alcohol dependence was absent in this group (Table 2, Table 3). Overall, 91% of individuals had received life-time diagnoses for two or more co-morbid conditions in addition to BPD, 56% were diagnosed with three or more additional co-morbid conditions, and 24% carried four or more co-morbid diagnoses in addition to BPD (Table 4). In this class, 62% were females and 38% were males. The mean age was 41 years with a standard deviation of 12.6. Class 2 (25% of the sample) was BPD with alcohol dependence and substance abuse/dependence. In this group, forty-seven percent had been diagnosed with four or more co-morbid conditions and seventy-five percent of individuals carried three or more diagnoses (Table 4). This class consisted of slightly more males (61%) than females (39%). Regarding obsessive compulsive disorder (OCD), panic disorder (PD), social phobia (SP), eating disorder (ED), and attention-deficit hyperactivity disorder (ADHD), Class 1 and Class 2 were not clearly differentiated (Table 1, Table 2, Table 3). The probability of endorsing psychotic symptoms (PSYCH) was higher in Latent Class 1 (41%) compared to Latent Class 2 (27%). Individuals in Class 3 (49% of the sample) were diagnosed with BPD, but their probability of endorsing any co-morbid condition was very low. Six percent of individuals were diagnosed with two co-morbid conditions; no individual carried more than two co-morbid diagnoses in addition to BPD. In 54% of individuals BPD was the only psychiatric diagnosis (Table 4). This class consisted of equal numbers of males and females and the mean age was the highest of all classes. In the Wald test significant differences in age and sex were present in all between-class comparisons, with the exception of the comparison between Class 1 and Class 2 on age. Since the assumption of heterogeneity in the sample with respect to co-morbidity was supported by the data, we were interested in testing if bipolar disorder with alcohol dependence might have different genetic risk factors than bipolar disorder without alcohol dependence. Taking heterogeneity with regard to co-morbid conditions into account would facilitate the identification of common genomic variants associated with specific sub-phenotypes.

**Table 2:** Probability of endorsing a co-morbid condition by latent class.

	Latent Class 1	Latent Class 2	Latent Class 3
**SUBA**	0.39	0.78	0.07
**OCD**	0.17	0.1	0.03
**PD**	0.37	0.31	0.12
**SP**	0.24	0.23	0.08
**ED**	0.12	0.10	0.03
**ADHD**	0.15	0.2	0.01
**ALCAB**	0.42	0	0.03
**ALCDEP**	0	1	0.18
**NIC**	0.30	0.47	0.08
**PSYCH**	0.40	0.29	0.17

All estimates were significant with two-tailed P-values<0.001 comparing the probability of endorsing an item versus not endorsing an item. SUBA, substance abuse; OCD, obsessive compulsive disorder; PD, panic disorder; SP, social and specific phobia; ED, eating disorder; ADHD, attention-deficit hyperactivity disorder; ALCAB, alcohol abuse; ALCDEP, alcohol dependence; NIC, nicotine dependence; PSYCH, psychotic symptoms (presence of hallucinations and/or delusions).

**Table 3:** Comparison of latent class profiles using odds ratios.

	LC1 compared to LC2		LC1 compared to LC3		LC2 compared to LC3	
	Estimate (S.E.)	P value*	Estimate (S.E.)	P value*	Estimate (S.E.)	P value*
**SUBA**	0.2 (0.1)	0.004	8.4 (3.9)	0.03	46.9 (19.9)	0.02
**OCD**	1.9 (0.6)	0.002	7.8 (6.8)	0.25	4.2 (3.4)	0.22
**PD**	1.3 (0.3)	0.000	4.4 (1.9)	0.02	3.5 (1.3)	0.006
**SP**	1.0 (0.3)	0.000	3.6 (1.7)	0.04	3.4 (1.4)	0.01
**ED**	1.1 (0.4)	0.001	5.1 (3.4)	0.14	4.5 (3.0)	0.14
**ADHD**	0.7 (0.2)	0.001	14.0 (13.5)	0.3	19.9 (18.7)	0.29
**ALCAB**	233 (340)	0.49	23.2 (25.0)	0.35	0.1 (0.2)	0.62
**ALCDEP**	N/A	N/A	N/A	N/A	N/A	N/A
**NIC**	0.5 (0.1)	0.000	4.9 (1.9)	0.01	10.2 (3.1)	0.001
**PSYCH**	1.6 (0.4)	0.000	3.4 (0.9)	0.000	2.1 (0.6)	0.000

*Two-tailed; LC, Latent Class; SUBA, substance abuse; OCD, obsessive compulsive disorder; PD, panic disorder; SP, social and specific phobia; ED, eating disorder; ADHD, attention-deficit hyperactivity disorder; ALCAB, alcohol abuse; ALCDEP, alcohol dependence; NIC, nicotine dependence; PSYCH, psychotic symptoms (presence of hallucinations and/or delusions).

**Table 4:** Co-occurrence of co-morbid conditions.

Diagnoses per individual in addition to BPD	Latent Class 1	Latent Class 2	Latent Class 3
**Four or more disorders**	24%	47%	0%
**Three or more disorders**	56%	75%	0%
**Two or more disorders**	91%	100%	6%

### Genome-wide association analysis

**Figure 3:**
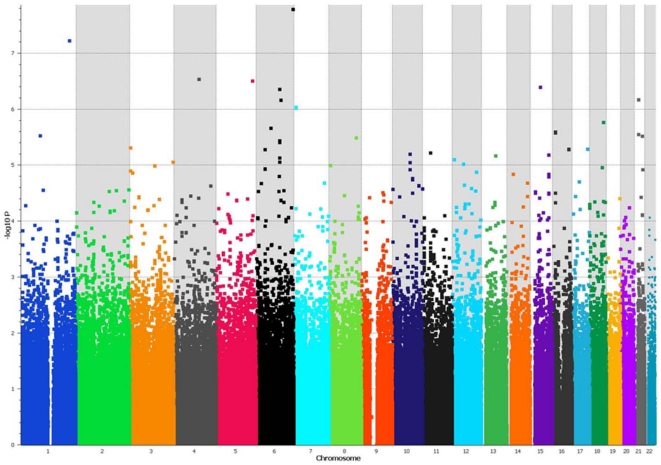
Genome-wide association analysis for Latent Class 1 under the recessive model. The Manhattan plot demonstrates the results of the genome-wide association analysis using the Latent Class 1 membership probability as phenotype. This subgroup of bipolar disorder patients was characterized by co-morbidity with substance abuse in 43% of individuals. In this group, 24% of individuals had been diagnosed with four or more co-morbid conditions in their lifetime. Alcohol abuse was prevalent in this patient group (52%), followed by substance abuse. Alcohol dependence was absent. Psychotic symptoms were found in 41% of individuals; about half of those had also used illegal substances. The x-axis depicts the position of the SNPs on the chromosomes with each chromosome shaded in a different color. The y-axis shows the −log10 p-value of the correlation trend test. The most significant finding was with the rare SNP rs1039002 on chromosome 6q27 (correlation trend test p = 1.7×10^−8^), followed by rs12563333 on chromosome 1q41 (correlation trend test p = 5.9×10^−8^). Within the chromosomal region indicated by SNP rs1039002 is the gene phosphodiesterase 10A. This gene has been implicated in the patho-physiology of psychosis in animal models.

**Figure 4:**
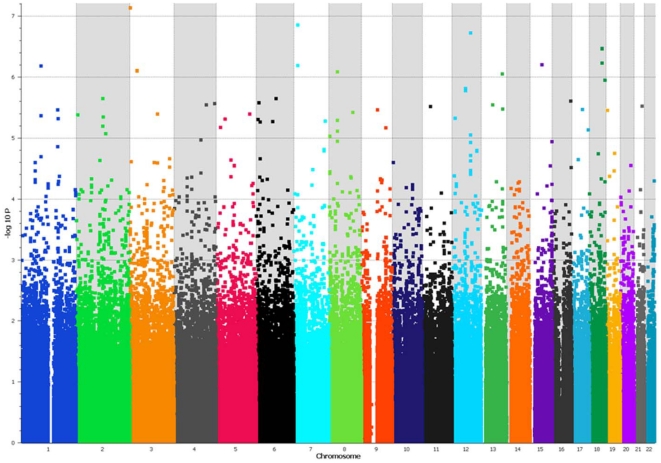
Genome-wide association analysis for Latent Class 2 under the recessive model. The Manhattan plot demonstrates the results of the genome-wide association analysis using the Latent Class 2 membership probability as phenotype. This subgroup of bipolar disorder patients was characterized by co-morbidity with alcohol dependence (100%) and substance abuse (80%). Forty-seven percent of individuals in this subgroup carried lifetime diagnoses of four or more co-morbid conditions. The x-axis depicts the position of the SNPs on the chromosomes, each chromosome shaded in a different color. The y-axis shows the −log10 p-value of the correlation trend test. The most significant association with Latent Class 2 membership probability was with SNP rs2727943 on chromosome 3p26.3 (correlation/trend test p = 3.0×10^−8^). This common intergenic variant is located between the genes contactin-4 precursor (*BIG-2*) and contactin 6 (*CNTN6*). Deletions in this chromosomal region have been previously associated with autism spectrum disorders.

The genome-wide association analyses with the most likely latent class membership as phenotype revealed three SNPs that reached or approached the level of genome-wide significance, two SNPs were associated with Latent Class 1 and one was associated with Latent Class 2 (Figure 3, Figure 4, Table 5, Table 6). In patients with co-morbidity in the absence of alcohol dependence (Latent Class 1) highly significant associations under the recessive model were found with SNP rs1039002 (p = 1.7×10^−8^, Bonf. P = 0.017, Perm. P = 0.023) and SNP rs12563333 (p = 5.9×10^−8^, Bonf. P = 0.04, Perm. P = 0.001). Rs1039002 is located on chromosome 6q27, 5′ of the processed transcript *RP11-252P19-001* (OTTHUMG00000015988) and close to the gene encoding phosphodiesterase 10A (*PDE10A*). Rs12563333 is located within the transcribed gene sequence *RP11-410C4.4* (OTTHUMG00000037350) on chromosome 1q41 immediately upstream of the gene encoding the MAP/microtubule affinity-regulating kinase 1 (*MARK1*) (HGNC:6896). Homozygotes for the minor allele of these SNPs were present only in Class 1 and absent in all other classes or the controls (Table 6). Two SNPs came close to the level of genome-wide significance. SNP rs9493867 on chromosome 6q23.2 within the gene encoding serine/threonine-protein kinase (*Sgk1*) (HGNC:10810) was associated with Latent Class 1 under the recessive model (p = 1.0×10^−7^). Homozygotes for SNP rs9493867 are absent in the HapMap sample. SNP rs13220542 located on chromosome 6q15 was associated under the dominant model (p = 9.0×10^−8^). This common variant is located 3′ to the gene encoding mitogen-activated protein kinase kinase kinase 7 (*MAP3K7*) (HGNC:6859). Both SNPs are located within gene regions.

**Table 5:** Results of genome-wide case control association analyses using latent class membership as phenotype.

Class	SNP	Chr.	Position	Gene	Corr. P	Bonf. P
**LC1**	rs1039002	6q27	166155457	RP11-252P19.1	1.7×10^−8^	0.01
	rs12563333	1q41	218724857	MARK1	5.9×10^−8^	0.04
**LC2**	rs2727943	3p26.3	1897973	intergenic	3.0×10^−8^	0.02

This table gives the SNP phenotype associations that reached or approached the genome-wide level of significance (after correction for multiple testing using the Bonferroni method). The position of the SNPs on the chromosomes is given in base pairs according to the Ensemble database, assembly GRCh37.p2, Feb 2009, Version 60.37e. SNP, single nucleotide polymorphism; LC1, Latent Class 1; LC2, Latent Class 2, Chr., chromosome; Corr P, p-value of the correlation-trend test, Bonf. P, Bonferroni corrected p-value.

**Table 6:** Genotype counts for SNPs with genome-wide significant associations.

SNP	MAF	Depend. AverageDD	Depend.AverageDd	Depend.Averagedd	GenotypeCountDD	GenotypeCountDd	GenotypeCountdd
**rs1039002**	0.06	0.95	0.14	0.14	4	222	1801
**rs12563333**	0.03	0.92	0.17	0.14	4	109	1921
**rs2727943**	0.15	0.36	0.12	0.12	46	498	1485

This table shows the minor allele frequencies for each SNP, as well as the dependent average latent class membership probability for each genotype. SNP, single nucleotide polymorphism; MAF, minor allele frequency; DD, homozygotes for the minor allele; Dd, heterozygotes for the minor allele; dd, homozygotes for the major allele.

BPD with high co-morbidity, substance abuse and alcohol dependence (Latent Class 2) was associated with SNP rs2727943 on chromosome 3p26.3 under the recessive model (p = 3.3×10^−8^, Bonf. P = 0.02, Perm. P = 0.03). SNP rs2727943 is a relatively common SNP (homozygotes for the minor allele were found in 3% of the HapMap samples) and it is not located in a gene region (Table 5, Table 6). The odds ratio of being a bipolar case for homozygote carriers of this SNP were 1.94; however, the odds ratio for being in Latent Class 2 compared to Latent Class 1, Latent Class 3 or controls was 4.9. No significant associations were found with Latent Class 3 or when all BPD cases combined were compared to the controls.

## Discussion

In recent years, there has been increasing recognition of co-morbid conditions in mental disorders. A deeper understanding of this phenomenon and its risk factors could improve treatment and prevention. Our results indicate heterogeneity in bipolar disorder patients with regard to co-morbidity consistent with emerging evidence from other studies. In this study we have explored possible genetic risk factors that might shed some light on underlying patho-mechanisms. Latent class analysis indicated the existence of three distinct subgroups of patients characterized by co-morbidity profiles, one group with predominantly substance abuse and/or psychosis, one group in which alcohol dependence prevailed, and one group with very low probability for any co-morbid conditions. Addressing this heterogeneity led to the identification of several highly significant associations with SNPs in genome-wide association analyses. Our results suggest that phenotype heterogeneity in BPD might indicate genetic heterogeneity. However, the interpretation of our findings poses several problems. Since genome-wide association analyses can, by design, only point to regions of the genome associated with a disease phenotype, the actual functional variants often remain elusive. Genome-wide association studies are also underpowered to replicate association with rare variants. Therefore, it is now commonly agreed upon that re-sequencing approaches are necessary to follow-up on genome-wide association studies and to identify the underlying causal variants.

The associations found in this study point to several interesting genes and chromosomal regions that might justify re-sequencing approaches in order to find the underlying functional variants. The genomic variant rs1039002, which was associated with a subgroup of bipolar patients with substance abuse and/or psychotic symptoms, is located close to a transcribed genomic sequence with unknown function. The product of the closest gene sequence with known function is phosphodiesterase 10A (*PDE10A*), a protein involved in the elimination of the intracellular signaling molecules cAMP and cGMP. The highest expression levels of this gene are found in heart, brain, kidney and testes. In the brain, expression is particularly high in the medium spiny neurons of the striatum. Inhibitors of this phosphodiesterase have shown therapeutic potential for the treatment of psychotic symptoms in schizophrenia, as well as the treatment of Parkinson's disease, Huntington's disease, addiction, and obsessive compulsive disorder. PDE10A inhibitors are now being tested in clinical trials [40], [41].

*RP11-410C4.4* is the closest expressed sequence to the SNP rs12563333, which is also associated with Latent Class 1 membership. This gene is located immediately 5′ to the serine/threonine-protein kinase gene *MARK1*. *MARK1* is highly expressed in brain and testes, with highest levels of expression found in the hippocampus. MARK1 phosphorylates microtubule-associated proteins and is involved in synaptic plasticity and dendritic trafficking [42]. Evidence for the involvement of this gene in autism has come from gene expression studies in postmortem brains, as well as genome-wide association studies [43]. Comparison of human *MARK1* with the mouse sequence showed significant differences and lack of conservation. It has therefore been hypothesized that *MARK1* could be involved in the development of higher cognitive functions that separate humans from mice [44]. However, it remains to be determined if rs12563333 is in linkage disequilibrium with any functional variants in *MARK1*.

Two SNPs that approached genome-wide significance are located in genes that are involved in response to environmental stresses, and therefore, might warrant further exploration. SNP rs9493867 on chromosome 6q23.2 is located within the gene serum/glucocorticoid regulated kinase 1 (*Sgk1*). This gene encodes a serine/threonine-protein kinase that is involved in the activation of potassium, sodium and chloride channels [45]. In rats it appears to be involved in memory consolidation, spatial learning, and cellular stress response through negative regulation of the SK1-JNK1-MEKK1 pathway [46], [47]. The second SNP is located in the 3′ region of the gene mitogen-activated protein kinase kinase kinase 7 (*MAP3K7*) on chromosome 6q15. This serine/threonine protein kinase is also involved in cell response to environmental stresses in related pathways through activation of MAPK8/JNK and the MAP2K4/MKK4 protein complex [48].

The most significant association with Latent Class 2 was within a region that has been found to be deleted in individuals with autistic features. SNP rs2727943 is located on chromosome 3p26.3 between the genes contactin-4 precursor (*BIG-2*) and the gene encoding the neural adhesion molecule contactin 6 (*CNTN6*). The protein products of these genes might play a role in the formation of axon connections in the developing brain [49], [50].

A limitation of our study is, foremost, the small sample size. Re-sequencing of the most significant regions in a larger sample of bipolar patients would be desirable in order to evaluate all variants in the identified genomic regions and to determine their functional consequences. The high genotype quality of the re-called and re-genotyped significant SNPs, the fact that the identified rare variants were present only in cases and not in controls and strong evidence for the therapeutic potential of PDE10A inhibitors might justify further follow-up studies. Our analysis indicates three major points. 1. Rare variants might be important pathogenic factors in BPD. Rare variants in gene regions were the most significant signals in our analysis, and these variants were exclusively present in cases and not in controls. In our small sample and without replication it is difficult to claim certainty about the disease association; however, cumulative evidence for the implication of the identified genomic regions could justify further investigation. 2. Since the associated variants were very rare, a genome-wide association design might not be the most appropriate approach for replication. Future studies could focus on re-sequencing of the chromosomal regions in a sample of BPD patients and controls in order to identify all rare and possibly coding variants in the region that might play a role in BPD pathophysiology. The fact that all of our associations were found in or near genes that have been implicated in psychiatric disorders and even in psychosis underlines the importance of these significant associations. 3. Taking the heterogeneity of the phenotype into account when performing disease association studies might increase the power of detecting significant associations.

In summary, our paper describes a novel multivariate approach to the phenotype of BPD. We identified rare variants in regions of interest that might warrant further studies. However, we were unable to find common variants with functional consequences associated with BPD. Focused or genome-wide re-sequencing might be important for the identification of genetic risk factors in bipolar disorder.

## Supporting Information

File S1 The supporting information provides the counts and proportions of the latent class solutions and the Bayesian information criteria, on which the latent class solution was selected. The QQ plot for the genome-wide association analysis under the recessive model is provided, followed by scatter plots for the genome-wide significant genotypes, first after the original genotyping, then after re-genotyping with the TagMan assay.
(DOC)
