# Supplementary material for: Genome-Wide Association Study in Bipolar Patients Stratified by Co-Morbidity
Source: PLoS One. 2011 Dec 21;6(12):e28477. doi: 10.1371/journal.pone.0028477 (PMC3244396; doi:10.1371/journal.pone.0028477)
Supplement: File S1 — The supporting information provides the counts and proportions of the latent class solutions and the Bayesian information criteria, on which the latent class solution was selected. The QQ plot for the genome-wide association analysis under the recessive model is provided, followed by scatter plots for the genome-wide significant genotypes, first after the original genotyping, then after re-genotyping with the TagMan assay. (DOC) [file pone.0028477.s001.doc]

**Supporting Information S1**

This supporting information provides the counts and proportions of the latent class solutions and the Bayesian information criteria, on which the latent class solution was selected. The QQ plot for the genome-wide association analysis under the recessive model is provided, followed by scatter plots for the genome-wide significant genotypes, first after the original genotyping, then after re-genotyping with the TagMan assay.

**Table S1A**

**Subgroups of bipolar patients based on co-morbidity**

| **Latent Class** | **Counts** | **Proportion** |
| --- | --- | --- |
| **Class 1** | 283 | 0.28 |
| **Class 2** | 246 | 0.25 |
| **Class 3** | 469 | 0.47 |

In order to explore heterogeneity in the phenotype, latent class mixture modeling was performed on a sample of bipolar patients using co-morbid conditions and psychotic symptoms as indicators for the latent subclasses. A three-class solution was selected as the best based on Bayesian information criteria. Latent Class 1 presented bipolar patients with low probability for substance abuse and alcohol dependence. Latent class 2 was bipolar patients with high probability of substance abuse and alcohol dependence, and Latent Class 3 were bipolar patients with generally low probability for any co-morbid psychiatric condition. The table shows the counts and proportions of these subclasses.

**Table S1B**

**Latent Class mixture model election criteria**

| **Class Solution** | **BIC** |
| --- | --- |
| **1** | 9303.476 |
| **2** | 9045.219 |
| **3** | 8931.080 |
| **4** | 8943.940 |

The Bayesian information criterion (BIC) was used to compare the latent class solutions. The solution with the smallest BIC was selected as the best.

**Figure S1A**

**Q-Q plot of BPD cases versus controls (recessive model)**

In order to ensure high quality genotypes, rigorous quality control measures were followed. First, we recalled the genotypes on the original signal intensity data using the CRLMM and BEAGLECALL algorithms. Then genotype calls with confidence<0.97 and/or MAF<0.001 were removed. The resulting genotype calls showed little evidence for batch effects and p-value inflation in association analyses as indicated by the QQ plots.

**Figure S1B**

**Scatter plots of genome-wide significant SNPs**

**SNP rs1039002 (SNP_A-1950036) (complete sample)**

**Figure 1SC**

**SNP rs1039002 confirmation (40 samples)**

**
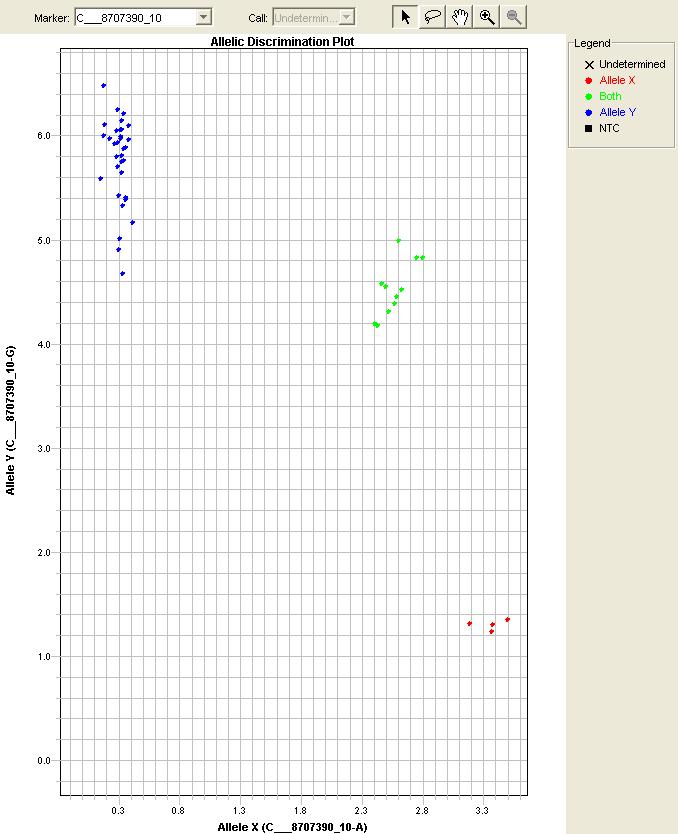
**

**Figure 1SD**

**SNP rs272794 (SNP_A-2206977) (complete sample)**

**Figure 1SE**

**SNP rs272794 confirmation (40 samples)**

**
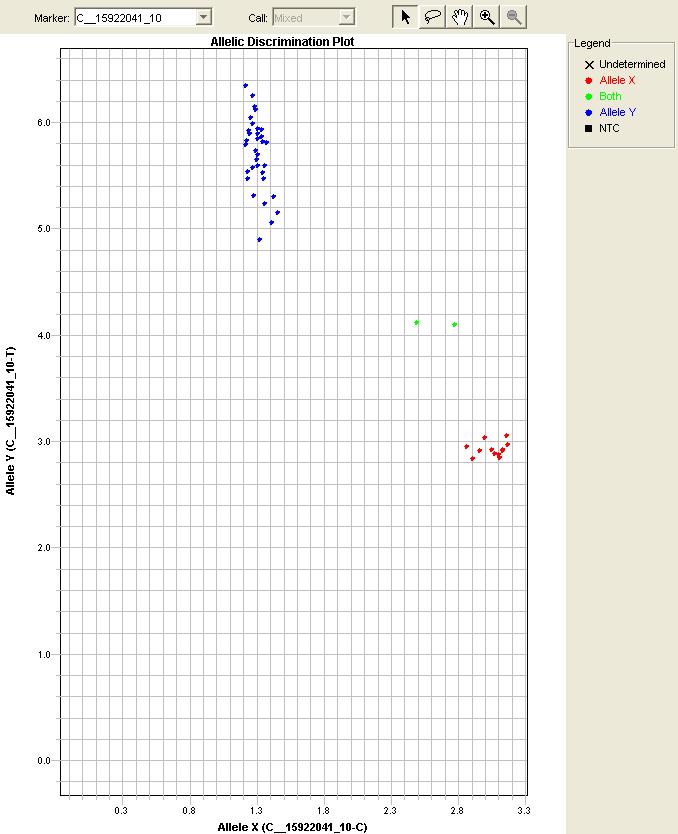
**

**Figure S1F**

**SNP rs12563333 (SNP_A-8356796) (complete sample)**

The original genotypes frequently demonstrated very poor clustering of alleles. Recalling the genotypes with the CRLMM algorithm and BEAGLECALL improved the confidence of the genotype calls. Scatter plots of SNP genotypes with genome-wide significant associations were examined by hand and then re-genotyped in 40 samples with TaqMan assays (TaqMan® SNP Genotyping Assay from Applied Biosystems run on the ABI 7900 Fast Real-Time PCR System (Life Technologies Corporation, Carlsbad, CA, USA) according to the published protocol (http://www3. appliedbiosystems.com/cms/groups/mcb_support/documents/generaldocuments/ cms_042998.pdf). Both assays showed good concordance. If the quality of the allele separation was satisfactory, scatterplots of SNPs that approached genome-wide significance were examined by hand only.
